# A Convolution Component-Based Method with Attention Mechanism for Travel-Time Prediction

**DOI:** 10.3390/s19092063

**Published:** 2019-05-03

**Authors:** Xiangdong Ran, Zhiguang Shan, Yufei Fang, Chuang Lin

**Affiliations:** 1 School of Computer Science and Technology, University of Science and Technology Beijing, Beijing 100083, China; fangyufei@sic.gov.cn; 2 Informatization and Industry Development Department, State Information Center, Beijing 100045, China; 3 College of Computer Science and Software Engineering, Shenzhen University, Shenzhen 518060, China; 4 Department of Computer Science and Technology, Tsinghua University, Beijing 100084, China; chlin@tsinghua.edu.cn

**Keywords:** convolutional neural network, attention mechanism, travel-time prediction

## Abstract

Deep learning approaches have been recently applied to traffic prediction because of their ability to extract features of traffic data. While convolutional neural networks may improve the predictive accuracy by transiting traffic data to images and extracting features in the images, the convolutional results can be improved by using the global-level representation that is a direct way to extract features. The time intervals are not considered as aspects of convolutional neural networks for traffic prediction. The attention mechanism may adaptively select a sequence of regions and only process the selected regions to better extract features when aspects are considered. In this paper, we propose the attention mechanism over the convolutional result for traffic prediction. The proposed method is based on multiple links. The time interval is considered as the aspect of attention mechanism. Based on the dataset provided by Highways England, the experimental results show that the proposed method can achieve better accuracy than the baseline methods.

## 1. Introduction

In recent years, the increase in vehicle transit and congestion on highways and urban road networks has led the changes on traffic conditions to uncertainty. Traffic prediction is still a key functional component and a research focus has been on intelligent transportation systems (ITS). Accurate and timely traffic prediction can help individual travelers, business sectors and government agencies make traffic decisions. These decisions may alleviate traffic congestion, reduce NOx compounds emissions and improve traffic operation efficiency. In various traffic indexes including traffic volume, travel-time, average speed, queue length and the severity of incidents, the delivery of travel-time is widely accepted as an index of ITS because travel-time is very intuitive and is easily understood [1,2,3].

Deep learning approaches, such as the recurrent state–space neural network (SSNN) [4], the long short-term memory neural network (LSTM NN) [5], the autoencoders (AES) [6], the deep learning approach with a sequence of tanh layers [7] and CNN-based methods [8,9,10] have been proposed for traffic prediction in recent years with their abilities to extract effectively features from traffic data. Experimental results show that these deep learning approaches achieve superior performance over other baseline methods. The generalization ability of CNN is implemented by providing the constraints in a task domain including local receptive fields, shared weights and spatial or temporal subsampling [11]. CNN-based methods of traffic prediction may improve the predictive accuracy by transiting traffic data to images and extracting features of the images. The task of traffic prediction differs from image processing task by providing constrains to the convolution component. The constrains in image processing task are the relationship between adjacent pixels, the constrains in traffic prediction task are the relationship between each historical traffic data point and the predicted value [12]. The convolution of the existing CNN-based methods for traffic prediction is implement by convoluting the adjacent pixels of traffic data [8,9,10]. While the global-level representation was proposed to provide the constrains in the task domain and directly capture the relationship [12], the experiments are only based on the single link but not based on multiple links. Meanwhile, the time intervals are not considered as aspects of traffic prediction in convolutional neural networks.

The attention mechanism has recently demonstrated success in a wide range of tasks by concentrating on the differences between input features to better extracting features when different aspects are considered [13,14,15]. The attention mechanism is usually implemented by constructing a glimpse network [13] that is a two-layer neural network. The attentive neural network is trained during the training of the proposed model.

**Figure 1:**
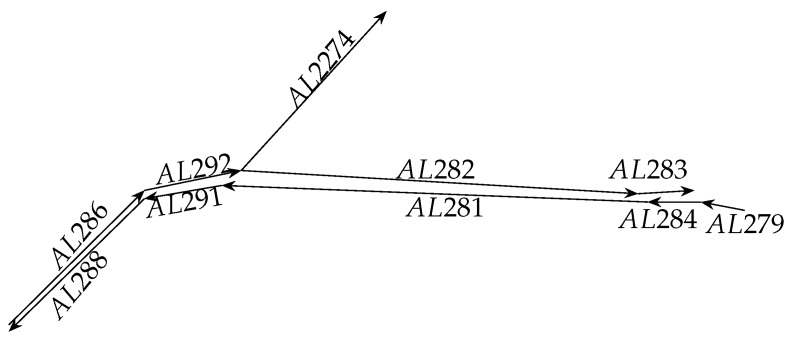
A section of road network that contains multiple links.

In this paper, we proposed the convolution component-based method with attention mechanism for travel-time prediction. The proposed method used the global level representation based on multiple links. For example, Figure 1 is a section of the road network. If the link AL282 will be predicted, the multiple links includes link AL282, AL283, AL2292, AL2274 and AL286. The time intervals are considered as the aspect of attention mechanism. Based on the dataset provided by Highways England, the proposed method is trained by using the back-propagation method and AdaGrad method to update the parameters of the proposed method. The experimental results show that the proposed method can achieve better predictive accuracy over baseline methods.

The main contributions of this paper can be summarized as follows.

- To directly extract features in multiple links, we substitute the global-level representation for the traditional local receive fields in the input sequences.
- We proposed the convolution component-based method with attention mechanism for travel-time prediction. The interval times are considered as the aspects of attention mechanism.
- Based on the dataset provided by Highways England, the proposed method is trained and the experimental results show that the proposed method can achieve better predictive accuracy over the baseline methods.

The rest of this paper is organized as follows. Section 2 describes the related works for traffic prediction. Section 3 describes the proposed method for travel-time prediction. Section 4 described the dataset provided by Highways England and the experimental setting based on the dataset. Section 5 shows and discusses the experimental results. Section 6 summarizes the conclusions and future work.

## 2. Related Work

Traffic prediction is still a key functional component and a research focus of ITS. Various traffic prediction methods have been proposed and improved the prediction accuracy in recent years. Deep learning approaches have been proposed for traffic prediction because of effectively extracting the features of traffic data. CNN-based methods may improve the predictive accuracy by transiting traffic conditions to images and effectively extracting features in the images. Despite the success of the CNN-based methods, such methods are clearly limited because they do not directly extract the features in multiple links and do not use interval times as an aspect.

### 2.1. Traffic Prediction Methods

Various methods have been proposed for traffic prediction in the past years. The target functions of these methods relate the explicative variables to the target variable, which are usually implemented by using statistical and machine learning techniques [4]. According to the implementation techniques of these methods, these methods can be grouped into two categories including statistical methods and machine learning methods. Time series models are the most typical statistical methods and usually include the autoregressive (AR), moving average (MA), autoregressive moving average (ARMA), autoregressive integrated moving average (ARIMA), seasonal autoregressive integrated moving average (SAIMA) and Kalman filtering method. The machine learning methods for traffic prediction usually includes the linear regression (LR), k-nearest neighbor regression (k-NNR), support vector regression (SVR), SSNN, LSTM NN, AES and CNN.

ARIMA (p, d, q) model combines AR model and MA model, where parameters p, d, and q are non-negative integers, p is the order (number of time lags) of the autoregressive model, d is the degree of differencing (the number of times the data have had past values subtracted), and q is the order of the moving-average model. ARIMA model is a generalization of ARMA model. The I in ARIMA model is the difference of the observation values to make the time series stationary. In [16], the ARIMA(0, 1, 3) was proposed for short-term traffic prediction. Based on the 166 data sets from three surveillance systems that are deployed on a freeway in Los Angeles, the experimental results show that the proposed model achieves better performance than MA model. In [17], an ARIMA (0, 1, 2) was proposed for traffic prediction. Based on the dataset on five major urban arterials, the experimental results show that the proposed model is effective in reproducing the original time series.

SARIMA model is ARIMA model with a seasonal component. In [18], Wold decomposition theorem states that any stationary time series can be decomposed into a deterministic series and a stochastic series. Based on the Wold decomposition theorem, it is hypothesized that a weekly seasonal difference between traffic data can yield a weakly stationary transformation and univariate traffic data streams can be modeled as a SARIMA process. The experiments are performed based on the dataset from two freeways and validated the theoretical hypothesis.

In [19], a prediction scheme based on Kalman filtering technique was proposed for traffic flow prediction. The method uses both historic data (previous two days flow data). Real time data on the day of interest was also attempted. Promising results were obtained with mean absolute percentage error (MAPE) of 10 between observed and predicted flows.

Researchers in the traffic prediction field have paid much attention to machine learning methods in recent years because of their ability of extracting features in traffic data.

In [20], it was proposed for travel-time prediction that a linear relation (LR) between the predicted travel-time $TT_{e}\left(t+\delta \right)$ and two naive predictors including the current status travel-time $T_{e}^{⋆}\left(t\right)$ and the historical mean travel-time $\mu_{TT}\left(t+\delta \right)$. Based on the dataset from 116 single-loop detectors, the proposed LR method outperforms the principal components method and the k-NNR method.

The accuracy of k-NNR may be improved by using a larger dataset [21]. K-NNR methods have some advantages over SARIMA model [22] and can avoid current time series data to lead inefficient predictions [23].

Support vector regression machine does not depend on the dimension of the input vectors space and may nonlinearly map input vectors to a high-dimension feature space to construct a linear decision surface. Thus, SVR will have advantages in high dimensionality space [24]. In [25], an SVR method with a radial basis function kernel (RBF) was proposed for traffic prediction and achieves better performance than the current-time predictor and the historical-mean predictor based on a highway traffic dataset. In [26], the online version of SVR was proposed for short-term travel-time prediction and achieves better performance than the Gaussian maximum likelihood, Holt exponential smoothing and the artificial neural network. In [27], the incremental SVR method was proposed for traffic flow prediction and achieves better performance than the back-propagation neural network.

Various neural networks have been proposed for traffic prediction because of their ability to extract features in traffic data. The advantages and disadvantages of individual deep learning methods are shown in Table 1. In [28], the Elman recurrent neural networks [29] were referred to as SSNN for travel-time prediction based on the traffic state-space formulation. In [6], AES method was proposed for traffic flow prediction and was trained in a greedy layerwise fashion. Based on freeway data from the freeway system in California, the experimental results show that the AES method outperforms the random walk (RW), SVR, RBF network and back-propagation neural network for traffic flow prediction. In [5], LSTM NN was proposed for traffic speed prediction. Based on the dataset on an expressway without signal controls, LSTM NN outperforms the ARIMA(2, 2, 2), SVM, Kalman filter [3], Elman NN, time-delay neural network (TDNN) [30] and nonlinear autoregressive with exogenous inputs neural network (NARX) [31]. In [7], the deep learning approach combines a sequence of tanh layers and a linear layer to capture the impacts of breakdowns, recoveries or congestion on traffic flow prediction. Based on the dataset from twenty-one loop detectors, the experimental results show that the deep learning method is effective for traffic flow prediction.

In [8,9,10], CNN-based methods were proposed for traffic prediction by transiting traffic data to images and extracting the adjacent relationship in the images. In [8], the CNN-based method was proposed for traffic speed prediction. In [9], the fusion of CNN and LSTM was proposed for passenger demand prediction. In [10], the CNN-based method with an errorfeedback RNN was proposed for traffic speed prediction. In [12], based on the single link, the global-level representation was proposed to directly capture the relationship in single link.

While various traffic prediction methods have improved the accuracy of traffic prediction, these methods have been evaluated under different datasets and thus it is difficult to say that one method is clearly superior over the other methods under any traffic conditions [6].

### 2.2. Convolution Neural Network

To improve the learning ability of neural networks, a more interesting scheme is to rely on the topology of the input data [11]. CNN was proposed to implement the scheme by combining three architectural ideas including local receptive fields, shared weights and spatial or temporal subsampling. By applying feature mapping and weight sharing, the learning ability of neural networks of recognizing handwritten zip codes was enhanced [34]. In [11], LeNet-5 was proposed for document recognition and achieves better performance than the baseline methods.

Three architectural ideas can be seen as that CNN integrates the constraints in the task domain into its architecture. The idea of providing constraints in task domain to the proposed method has also been widely applied in many other fields. In [33], the convolutional LSTM extends the fully connected LSTM to have convolutional structures in both the input-to-state and state-to-state transitions. The experimental results show that the convolutional LSTM outperforms the fully connected LSTM for precipitation prediction. In [32], the convolutional architecture with piecewise max pooling utilizes all local features to perform relation extraction globally. The experimental results show that the proposed method achieves better performance than the baseline methods for relation extraction. In [12], based on the single link, the global-level representation was proposed to directly capture the relationship in single link. In this paper, we use the global-level representation to integrate the constraints in multiple links.

### 2.3. Attention Mechanism

Attention mechanism has recently succeeded in many tasks including image classification [13], neural machine translation [14], multimedia recommendation [15], because it may focus on the effective parts of features when different aspects are considered. The attention mechanism is usually implemented by using a neural network based on the corresponding task. In [13], attention mechanism was proposed to address the problem of enormous computation cost in CNN. In [14], attentional mechanism selectively focuses on the effective parts of input sentences during translating to improve the accuracy of machine translation. In [15], the two-layer attention mechanism is proposed to adaptively extract the implicit feedback. In this paper, the attention mechanism is proposed to use interval times as aspect for traffic prediction.

In [35], the convolutional block attention module was proposed for object detection. Given an intermediate feature map, the proposed module sequentially infers attention maps along two separate dimensions, channel and spatial, then the attention maps are multiplied to the input feature map for adaptive feature refinement. The experimental results show that the proposed method outperforms all the baselines. The attention mechanism in our work is similar to the channel attention in [35].

## 3. Methodology

### 3.1. Neural Network Architecture

In the paper, the traffic prediction task is described as follows. Given the traffic data on some links, the traffic time within time interval (t+δ) on the selected link will be predicted. Where *T* is the current interval time and δ is some span of interval time.

**Figure 2:**
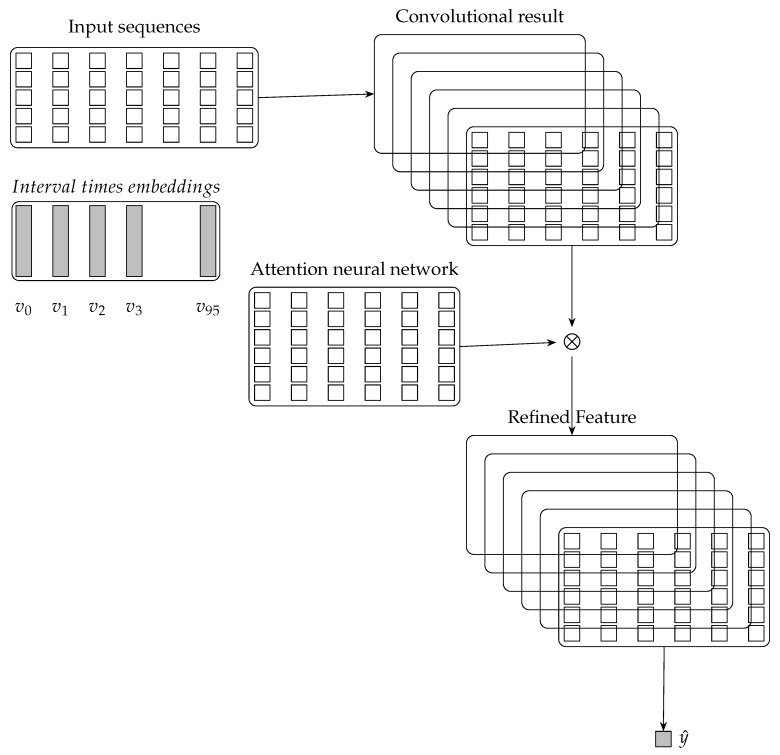
Architecture of the convolutional neural networks wich attention mechanism for travel-time prediction.

The architecture of the proposed method for traffic prediction is illustrated in Figure 2. The input sequences including attribute travel speed and attribute interval time will be fed into the architecture one by one. The predicted travel-time will be computed based on the predicted travel speed and the length of the selected link. Based on the input sequences from multiple links, the global level representation provide the constraints in the task domain for our proposed method [12]. The convolution extracts features from the global-level representation of multiple links for traffic prediction. The attention mechanism is a glimpse network and is implemented by a two-layers neural network. The attention mechanism adaptively select a sequence of regions and only process the selected regions when interval time aspects are considered. The convolutional results will be flattened and fed to a single layer neural network to generate the predicted value.

Interval time is 15 min in the given database, thus there are 96 interval times per day. 96 is the length of interval times embeddings that is a vector and will be learned during model training. Each item of the interval times embedding represent one interval time.

The architecture of our proposed method includes the interval times embeddings, the input sequences component, the global-level representation component, the convolution component, the attention mechanism component and the out predictor.

### 3.2. Input Sequence

Selecting attributes from the given dataset is the important step of data preparation procedure of data process [36]. The selected attributes in the CNN based methods are usually the same as the attributes to be predicted [8,9,10]. While the success of these CNN-based methods, the attribute interval time is not selected as the attribute of these CNN-based methods that represents the temporal-level structure of the traffic data. In our work, the attribute interval time is selected.

Let current interval time be *t* and the length of the input sequence be 7, then the input sequence in Figure 1 is described in Equations (1) and (2). Equation (1) denotes the data from the link to be predicted. Equations (2) denotes the data from other links including on-ramps, off-ramps or other adjacent links, *i* is the index of the multiple links, where *x* is $\left[\begin{array}{c} CF\\ SF \end{array}\right]$. SF is the attribute interval time regarding the temporal-level structure and CF is the attribute travel-time regarding the content. Where the subscripts {t−6,t−5,t−4,t−3,t−2,t−1,t} are interval times. The predicted value is at interval time t+δ and δ denotes time lag that is the interval time of the predicted value and current interval time.

(1) $$s:x_{t-6},x_{t-5},x_{t-4},x_{t-3},x_{t-2},x_{t-1},x_{t}$$

(2) $$s^{i}:x_{t-6}^{i},x_{t-5}^{i},x_{t-4}^{i},x_{t-3}^{i},x_{t-2}^{i},x_{t-1}^{i},x_{t}^{i}$$

### 3.3. Convolution

The learning ability of CNN may be enhanced by providing constraints in the task domain [34], in which the constraints in the task domain include the local receptive fields and the shared weights. In our work, the global-level representation was proposed that each *x* in the input sequence convolutes $x_{t-1}$ in the input sequence, respectively [12]. The global-level representation is the substitute for the local receptive fields of the convolution component to apply the constraints in traffic prediction task. The constraints is that each historical data point in the input sequence directly effect the predicted value [12].

The convolution is a linear transformation described in Equation 3. Where $X\left(i,j\right)=\left(x_{t-i},x_{t}\right)$ is one global-level representation. i∈(1,…,L) and *L* is the length of the input sequence. *t* is the length of training dataset or testing dataset. Filter $W_{k},k\;\in \left(1,\ldots ,n_{1}\right)$ is the shared parameter matrix and hyperparameter $n_{1}$ is the number of the filters. Matrix $C\in R^{n_{1}\times t}$ denotes the convolutional result. C(i;j) denotes the convolutional result of global-level representation X(i,j). In Equation (3), weight matrix $W_{k}$ is shared across all times.

(3) $$\begin{array}{c} C\left(i,j\right)=W_{k}X\left(i,j\right)\;1<i\leq L\;0<j\leq t\;k\in \left(1,\ldots ,n_{1}\right) \end{array}$$

### 3.4. Attention Mechanism

**Figure 3:**
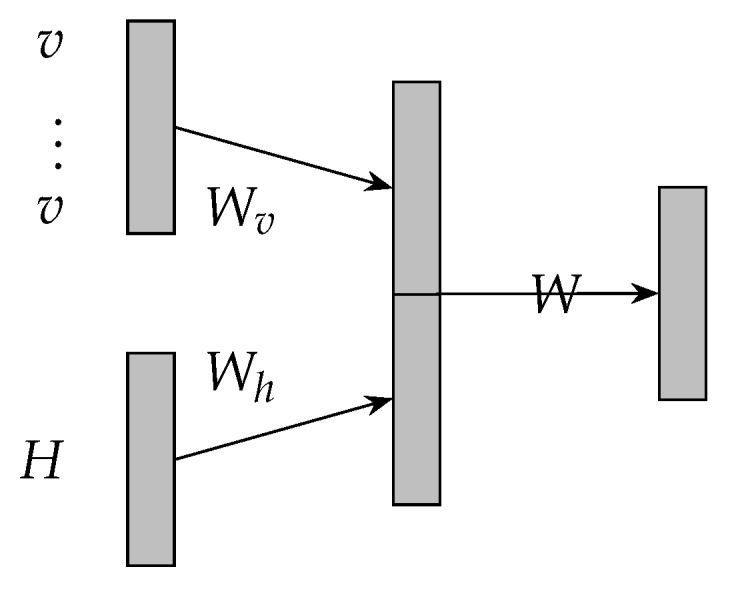
Architecture of attentive neural network.

Attention mechanism is implemented by constructing a glimpse network according to the corresponding tasks and has recently demonstrated success [13,14,15,35]. The attention mechanism in literature [13] is over the input images and adaptively select a sequence of regions of the input images and only process the selected regions. In our work, the attention mechanism is over the convolutional results and adaptively select a sequence of regions of the convolutional results and only process the selected regions. The attention mechanism of the proposed model is illustrated in Figure 3 and its transition functions are described in Equations (4)–(6). Where matrix *H* denotes the convolutional results illustrated in Figure 2. $v_{a}$ is the embedding of the interval time. Vector α denotes the attention weights to matrix *H*. Matrix *r* denotes the element-wise multiplication between matrix *H* and α. Specifically, based on an interval time aspect, attention mechanism allows the proposed model to attend over matrix *H* based on an interval time and may effectively extracts the features in matrix *H*.

(4) $$M=tanh\left[\begin{array}{c} W_{h}H\\ W_{v}v_{a}⊗e_{n} \end{array}\right]$$

(5) $$\alpha =w^{T}M$$

(6) $$r=H\alpha^{T}$$

### 3.5. Output

The predictor is a single layer fully-connected neural network and is described in Equation (7). Where matrix $W_{2}\in R^{l\times n_{1}}$ denotes the parameter of the predictor. The predictor receives the output of attention mechanism *r* to compute the predicted travel-time haty, which is the travel-time across the given link within time interval T+δ.

(7) $$\begin{array}{c} \hat{y}=W_{2}r \end{array}$$

### 3.6. Model Training

The parameters of the proposed method is $\theta :\left\{W_{k},W_{2},W_{h},W_{v},w,V_{a},n_{1},l\right\},k\in \{1,\ldots ,n_{1}\}$. Where $\left\{W_{k},W_{2},W_{h},W_{v},w,V_{a}\right\}$ are randomly initialized and updated during the training of the proposed method. $n_{1}$ is a hyper-parameter and is the number of the kernels of the convolution compnent. *l* is the length of the input sequence. The proposed method is trained by using the back-propagation method. The cost function of the proposed method is described in Equations (4)–(6). Where $S=\{\left(s_{i},y_{i}\right)\}$ denotes the training examples. $\left\{s_{i}\right\}$ denotes the input sequences. $y_{i}$ denotes the travel-time within time interval T+δ. $\hat{y_{i}}$ denotes the predicted travel-time within time interval T+δ.

The mini-batch gradient descent method is used to compute the cost function and the partial derivative of parameters θ. AdaGrad is used to update parameters θ. AdaGrad is described in Equation (6), where ε is a smoothing term that avoids division by zero (usually on the order of $1\times 10^{-8}$). AdaGrad performs the larger updates for infrequent parameters rather than frequent parameters. Thus, the need to manually tune the learning rate of gradient descent methods is eliminated and the robustness of gradient descent methods is greatly improved [37]. Theano is used to develop the code of the proposed method. The proposed method is trained on a 4-G GPU computer with CUDA [38] in which CNMeM is enabled with an initial size by using 80.0% of the memory.

We choose hyperparameters n1 based on validation set, adjust parameter matrices $\left\{W_{1},W_{2},W_{h},W_{v},w,V_{a}\right\}$ based on training set and evaluate the accuracy of the proposed method based on testing set.

(8) $$\begin{array}{c} J\left(\theta \right)=\sum^{S}\frac{1}{\left|S\right|}\|{\hat{y}}_{\theta }\left(s_{i}\right)-y_{i}{\|}^{2} \end{array}$$

(9) $$\begin{array}{cc} G_{t,i} & =\sum^{t}\nabla_{\theta_{i}}{J(\theta )}^{2} \end{array}$$

(10) $$\begin{array}{cc} \theta_{t+1,i} & =\theta_{t,i}-\frac{\eta }{\sqrt{G_{t,i}}+\varepsilon }\cdot \nabla_{\theta_{i}}J(\theta ) \end{array}$$

### 3.7. Dataset and Task Definition

We evaluate the performance of the proposed method based on the dataset provided by Highways England [39]. Highways England have maintained and improved the motorways and major A roads in the England. The dataset for our experiment is described in Table 2, where AverageJT is the travel-time that is taken by vehicles to across LinkRef within TimePeriod. TimePeriod denotes time interval that vary from 0 to 95 and is 15 min.

The database of the links spans from 1 March 2015 to 31 March 2015. We divide the dataset into three parts including the training set, the validation set and the testing set, which contain data from 1 March to 27 March (approximately 87.1%), March 28 (approximately 3.2%) and 29 March to 31 March (approximately 9.7%), respectively.

**Input Sequence and Its Output Value**: Let we predict the travel-time across AL282 within time interval t+δ. The input sequence is described in Equation (11), where δ is a time lag and CF is the travel-time attribute.

(11) $$S:\left[\begin{array}{ccccccc} x_{t-6}^{\mathrm{AL}282} & x_{t-5}^{\mathrm{AL}282} & x_{t-4}^{\mathrm{AL}282} & x_{t-3}^{\mathrm{AL}282} & x_{t-2}^{\mathrm{AL}282} & x_{t-1}^{\mathrm{AL}282} & x_{t}^{\mathrm{AL}282}\\ x_{t-6}^{\mathrm{AL}283} & x_{t-5}^{\mathrm{AL}283} & x_{t-4}^{\mathrm{AL}283} & x_{t-3}^{\mathrm{AL}283} & x_{t-2}^{\mathrm{AL}283} & x_{t-1}^{\mathrm{AL}283} & x_{t}^{\mathrm{AL}283}\\ x_{t-6}^{\mathrm{AL}292} & x_{t-5}^{\mathrm{AL}292} & x_{t-4}^{\mathrm{AL}292} & x_{t-3}^{\mathrm{AL}292} & x_{t-2}^{\mathrm{AL}292} & x_{t-1}^{\mathrm{AL}292} & x_{t}^{\mathrm{AL}292}\\ x_{t-6}^{\mathrm{AL}286} & x_{t-5}^{\mathrm{AL}286} & x_{t-4}^{\mathrm{AL}286} & x_{t-3}^{\mathrm{AL}286} & x_{t-2}^{\mathrm{AL}286} & x_{t-1}^{\mathrm{AL}286} & x_{t}^{\mathrm{AL}286}\\ x_{t-6}^{\mathrm{AL}2274} & x_{t-5}^{\mathrm{AL}2274} & x_{t-4}^{\mathrm{AL}2274} & x_{t-3}^{\mathrm{AL}2274} & x_{t-2}^{\mathrm{AL}2274} & x_{t-1}^{\mathrm{AL}2274} & x_{t}^{\mathrm{AL}2274} \end{array}\right.$$

(12) $$\hat{y}=x_{t+\delta }^{\mathrm{AL}282}.CF$$

## 4. Experimental Setting

**Normalization**: To avoid calculation overflow during the training process and better capture the nonlinear relationships, CF and SF are rescaled to (−1, 1) because (−1, 1) is the range of the activation function in the proposed method.

### 4.1. Evaluation Metrics

To evaluate the performance of the predictive methods, we adopt three performance indices including the mean absolute error (MAE), the mean absolute percentage error (MAPE) and the RMS error (RMSE). These indices are stated as Equations (13)–(15), respectively. Where *S* denotes the input sequences. *i* is the index input sequence in *S*. $y_{i}$ denotes the observed value and $\hat{y_{i}}$ denotes the output value.

(13) $$\mathrm{MAE}=\frac{1}{\left|S\right|}\sum_{i=1}^{\left|S\right|}\left|\hat{y_{i}}-y_{i}\right|$$

(14) $$\mathrm{MAPE}=\frac{1}{\left|S\right|}\sum_{i=1}^{\left|S\right|}\frac{|\hat{y_{i}}-y_{i}|}{|y_{i}|}$$

(15) $$\mathrm{RMSE}={\left[\frac{1}{\left|S\right|}\sum_{i=1}^{\left|S\right|}{\left(\hat{y_{i}}-y_{i}\right)}^{2}\right]}^{\frac{1}{2}}$$

### 4.2. Hyperparameters

**Figure 4:**
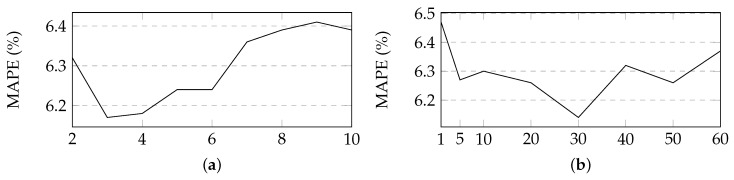
Variety of the MAP with parameters *l* and $n_{1}$ in the proposed method. (**a**) Size of an input sequence. (**b**) Number of the kernels of the convolution compnent.

In this section, we experimentally determine hyperparameter $\left\{n_{1},l\right\}$ based on the validation set. *l*, the length of an input sequence and $n_{1}$, the number of the kernels of the convolution compnent. The hyperparameter values is determined in a certain range as follows: *l* in {2,…,10} and $n_{1}$ in {1,5,10,20,…,60}. Figure 4 describes the variety of the MAPE with parameters *l*, $n_{1}$ increasing. The optimal values of *l* and $n_{1}$ are 3 and 30, respectively. The optimal learning rate of the parameters for AdaGrad, η, is 0.1. Table 3 describes the hyperparameter values that are used in our experiments.

### 4.3. Baseline Methods

Eight methods are selected as the baseline methods to evaluate the performance of our proposed method. These methods include SARIMA, LR, k-NNR, SVR, SSNN, LSTM NN, CNN and AEs. In our experiments, we predict the travel-time within time interval t+δ on link AL282 where lag δ is one time interval.

The LR method in our experiments is based on a publication by Rice and Zwet [20] in which the predicted travel-time is the linear regression of the travel-time within time interval *t* and the mean travel-time within historical time interval t+δ.

The SARIMA method in our experiments is based on the contributions in the literature [18] in which the input sequences are considered as a SARIMA $\left(1,0,1\right){\left(0,1,1\right)}_{S}$ process. We let hyperparameter *S* be 96 because there are 96 time intervals per day in the our dataset.

The Kalman filter method in our experiment is based on the contributions in literature [19]. The traffic values observed on the previous day was used in place of the observation for correcting the apriori estimate.

In the k-NNR method in our experiments, the predicted value is a weighted average value of the k-NNs in which the time intervals are before time interval t+δ [22]. The weights that are assigned to the k-NNs are usually accomplished by using a distance scheme or a uniform scheme. The distance scheme assigns different weights, usually the inverse of their distance to the predicted data point, to the contributions of the k-NNs. The uniform scheme assigns the same weights to the contributions of the k-NNs.

The SVR method with an RBF kernel is based on the contributions in literature [25]. The SVR method in our experiments uses three types of kernels, namely, a linear kernel (linear), a polynomial kernel (poly), and an RBF kernel.

The SSNN method is based on the contributions in literature [4] in which the connection between the internal states and the context layers is fixed at 1.0 and the values of the nodes in the context layer are initially set to 0.5. The connection and the values are not subject to adjustment in the training process. In our experiments, we let the number of nodes in the hidden layer and the output layer be 10. We let the length of the input sequence and the unfolded size of SSNN be 12.

The LSTM NN is based on a publication in literature [5]. In our experiments, we let the unfolded size of the LSTM NN be the same as literature. The AEs method in our experiments is based on a publication in literature [6]. The CNN1 method in our experiments is based on a publication in literature [8]. The CNN2 method in our experiments is based on the publication in literature [12].

In addition, we parameterize the baseline methods as described in the original literatures, in which the baseline methods have been tuned for other datasets. This hints that they perhaps perform considerably better on this dataset if they also gets tuned for it. For future work, we will turn the parameters of the baseline methods to perform a further comparison.

## 5. Experimental Results

### 5.1. Exploring the Spatiotemporal Relationship

The CNN-based method is effective because there is exist spatiotemporal relationship in multiple links. We demonstrate the relationship by using Pearson correlation in Equation (16), where *X*, *Y* denote two random variables with the same number of observations. In literature [9], the Pearson correlation is also used to explore the spatiotemporal relationship of the variables in short-term passenger demand prediction. We calculate the Pearson correlations between the predicted travel-time at interval time *t* in link *i* and the history travel-time at time interval t−k in link *j*. Where (*i* = AL282, j∈ {AL286, AL292, AL2274, AL282, AL283}) or (*i* = AL281, j∈ {AL288, AL291, AL281, AL284}), k∈{1,2,3,4,5,6,7}.

(16) $$\begin{array}{cc} Corr(X,Y) & =\frac{E(XY)-E(X)E(Y)}{\sqrt{E\left(X^{2}\right)-E^{2}\left(X\right)}\sqrt{\left(Y^{2}\right)-E^{2}\left(Y\right)}} \end{array}$$

**Figure 5:**
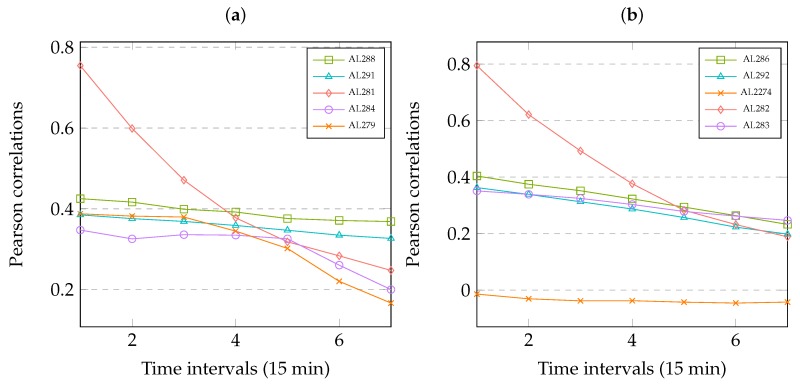
Correlations in multiple links. (**a**) Time intervals on Link AL281. (**b**) Time intervals on Link AL282.

Figure 5 shows the relationship in multiple links that is correlations between the dependent variable (the to-be-predicted travel-time at interval time *t* on Link *i*) and the explanatory variables (the observed travel-time at interval time t−k on Link *j*).

It can show that there are strong temporal correlations among dependent variable and the explanatory variables, which drop gradually with the increase of time intervals. On the other hand, the variables with shorter spatial distance have strong correlations, but the variables with longer spatial distance are also correlated with the to-be-predicted travel-time to some extent.

### 5.2. Performance with Time Variation

**Figure 6:**
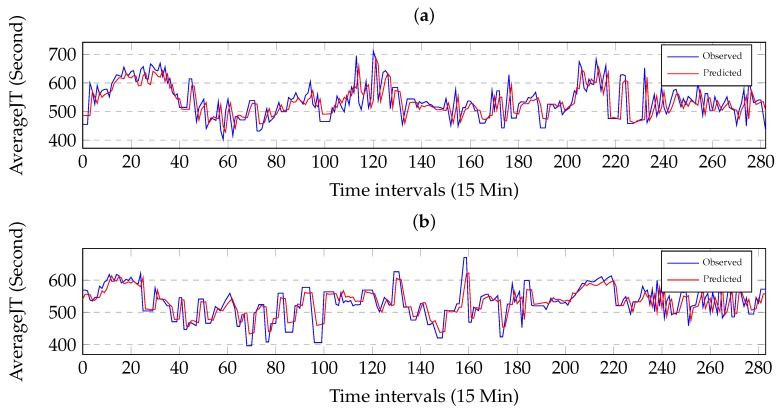
Effectiveness of the prediction under the different traffic conditions. The horizontal axis is time interval and the vertical axis is travel-time. (**a**) Comparison between the observed values and the predicted values on Link AL282. (**b**) Comparison between the observed values and the predicted values on Link AL281.

We study the prediction performances of the proposed method during 3 day under different traffic conditions where the time lag is one time interval. Figure 6a illustrates the comparison between the observed values and the predicted values on link AL282. Figure 6b illustrates the comparison between the observed values and the predicted values on link AL281. The comparison can be well visualized when zooming into a finer grade. Figure 6 shows that the observed values and the predicted values are in good agreement and the proposed method is effective. Figure 6 shows that the proposed method may capture the sudden changes in travel-time and has a tendency to underestimate the future traffic condition. Specially, the proposed method may capture the sudden regime changes from free flow to congestion and then the recovery regime.

**Figure 7:**
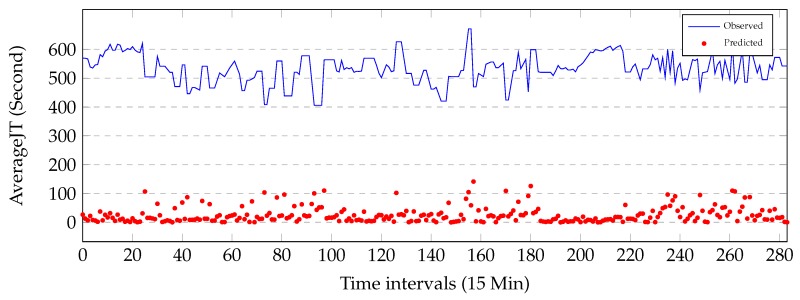
Absolute values of the residuals against the observed data during 3 days on link AL281. The horizontal axis is time intervals and the vertical axis is travel-times.

Figure 7 illustrates that the absolute values of the residuals (red circles) against the observed data (blue line) during 3 days on link AL281. We can see that the performance of the proposed method is not uniform through days. Several large errors are observed at when regime changes from free flow to congestion and starts to recover back to free flow. Highest residuals are observe at when travel-time regime changes from one to another.

**Figure 8:**
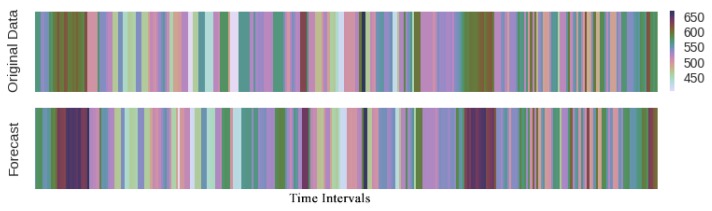
Heat plots of travel-time prediction during 3 days. The horizontal axis is time intervals.

Heat hots Figure 8 is another visual way to interpret the results of prediction where the deeper color implies the longer travel-time. The congestion propagation on traffic network led traffic prediction to complex. For example, we consider a stretch of highway and assume a bottleneck, it is expected that the end of the queue will move from the bottleneck downstream. Both the head and tail of the bottleneck sometimes move downstream together. Figure 8 shows that the proposed method properly may capture both forward and backward shock wave propagation on link AL281 during 3 days.

**Figure 9:**
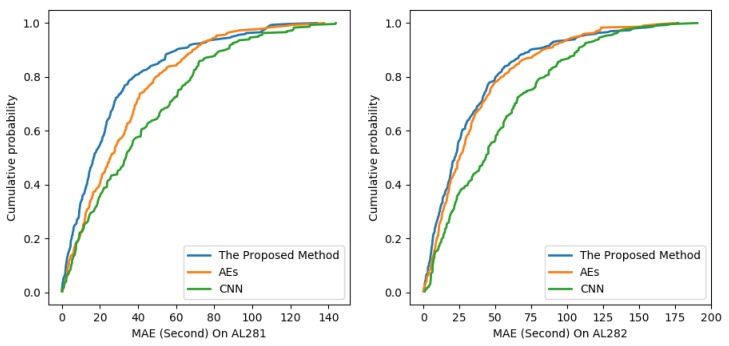
Cumulative distribution of the absolute predictive errors on link AL281 and link AL282.

Figure 9 plots the cumulative distribution functions of the absolute prediction errors on links AL281 and AL282 to demonstrate the statistical property of the proposed method, CNN and AES, respectively. The time interval is 15 min. Figure 9 shows that the prediction error of the proposed method is smaller than CNN and AEs on link AL281 and link AL282. In summary, the experimental results testified the effectiveness of the proposed method for travel-time prediction.

### 5.3. Overall Performance

To compare the proposed method with the baseline methods, we perform the experiments that predict the travel-time across link AL282 within time interval T+1 based on multiple links AL286, AL292, AL2274 AL282 and AL283. We perform the experiments that predict the travel-time across link AL281 within time interval T+1 based on multiple links AL288, AL291, AL281, AL284 and AL279. The length of link AL282 is approximately 15.64 km and the length of link AL281 is approximately 16.00 km. The multiple links is illustrated in Figure 3.

Table 4 shows the experimental results across link AL282. The experimental results show that the proposed method achieves better accuracy than the baseline methods. Table 5 shows the experimental results across link AL281. The experimental results shows that the proposed method achieves better accuracy that the baseline methods.

**Discussion of Results**: Prediction errors may usually be decomposed into two subcomponents including bias error attributed to erroneous assumptions and variance error attributed to the sensitivity to small fluctuations. High bias denotes that the methods miss some relevant relations between the features and the predictive outputs (under fitting). Low bias but high variance denotes that the random noises in the training dataset are transformed by the methods rather than the intended outputs (over fitting) [40]. Based on the multiple links illustrated in Figure 3, Table 4 and Table 5 show that the proposed method outperforms all baseline methods in terms of bias error and the variance of the proposed method has the same order of magnitude as the NN-based methods. Thus, the proposed method has no under-fitting or over-fitting problems and is an effective approach for travel-time prediction.

**Figure 10:**
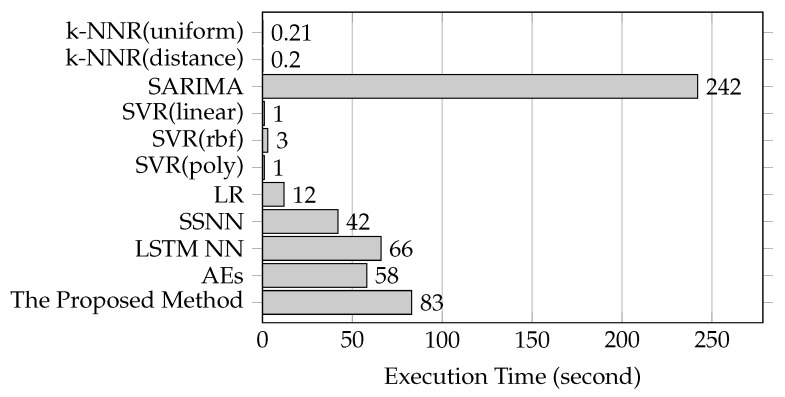
Time costs of the proposed method and the baseline methods.

**Time Costs**: Figure 10 shows the execution times spent by the proposed method and the baseline methods based on link AL282. According to the time costs, these methods are classified into three groups including SARIMA method, NN-based methods and the remaining methods. SARIMA method takes the most time to execute, the NN-based methods take the moderate time to execute and the remaining methods take the least time to execute. The proposed method is within a reasonable boundary of time consumption.

### 5.4. Performance for the Individual Links

**Figure 11:**
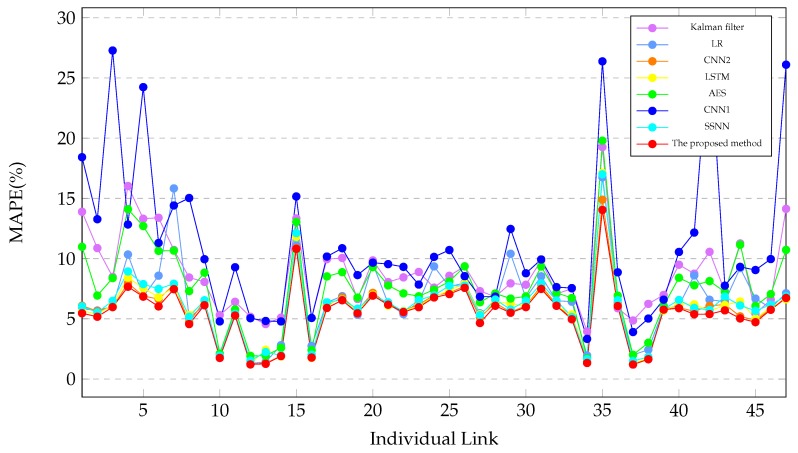
Experimental results of the proposed method and the baseline methods on individual links.

To validate the performance of the proposed method, we perform the experiments on every road, which are AL344, AL3276, AL444, AL2878, AL2869, AL2871, AL2861, AL2853, AL2850, AL2852B, AL2852A, AL286, AL292, AL283, AL278, AL270, AL265, AL261A, AL258A, AL256, AL2282, AL248, AL241, AL242, AL236, AL2292, AL2295, AL237, AL243, AL240, AL254, AL257A, AL267, AL272, AL279, AL284, AL291, AL288, AL2851A, AL2851B, AL2849, AL298, AL2862, AL343, AL2872, AL2870 and AL2879. There are 47 links. For each link, we cluster five adjacent links as multiple links for training and testing. we perform the experiments to predict travel-time across these links. The baseline methods include LR, LSTM, SSNN, AES, CNN1 [8] and CNN2 [12]. The experiment results are described in Table 6 and in Figure 11. Where the interval time is 15 min.

Table 6 and Figure 11 shows the MAPE of these links. Table 6 and Figure 11 shows that the proposed method outperforms the baseline methods on these links. Table 6 and Figure 11 indicates that the prediction errors of different links vary greatly. The predictability of links #10, #12, #13, #14, #16, #34, #37 and #38 ( AL2852, AL286, AL292, AL283, AL270, AL272 and AL291 ) are much better, the predictability of links #17, #30, #34 and #44 (AL265, AL240, AL272 and AL343) are much poorer.

## 6. Conclusions and Future Work

In this paper, we propose the convolution component-based method with attention mechanism for traffic prediction. The global level representation is used based on multiple links. The time intervals is considered as the aspect of attention mechanism. Based on the dataset provided by Highways England, the experimental results show that the proposed method can improve the predictive accuracy than the baseline methods.

The proposed method still exist a weakness that it have the bigger absolute prediction errors at some time intervals. Improving the weakness may be conducted as a future work. Another future work is providing a better substitute for the predicted travel-time in the input sequences.

## Figures and Tables

**Table 1:** Advantages and disadvantages of individual deep learning methods.

Methods	Advantages and Disadvantages
SSNN	Use shared internal state (memory) to process sequences of inputs and exists the issue of back-propagated error decay through memory blocks [4].
LSTM NN	Can overcome the issue of back-propagated error decays through memory blocks [5].
AES	Spatial and temporal correlations are inherently considered [6].
CNN	Temporal correlations are inherently considered [12,32,33]. Based on the topology of the input data, three architectural ideas are proposed including local receptive fields, shared weights and spatial or temporal subsampling [34].

**Table 2:** Description of the dataset provided by Highways England.

LinkRef	Date	TimePeriod (0–95)	AverageJT	LinkLength (km)
AL282	2015-03-01	0	642.56	15.64
AL282	2015-03-01	1	603.71	15.64
…	…	…	…	…
AL292	2015-03-01	0	126.03	3.92
AL292	2015-03-01	1	130.52	3.92
…	…	…	…	…
AL2274	2015-03-01	0	337.40	9.72
AL2274	2015-03-01	1	337.40	9.72
…	…	…	…	…
AL286	2015-03-01	0	271.83	7.98
AL286	2015-03-01	1	272.69	7.98
…	…	…	…	…

**Table 3:** Hyper parameters value used in our experiments.

Hyperparameter	*l*	$n_{1}$	η
**Value**	3	30	0.1

**Table 4:** Experimental results of the proposed method and the baseline methods on link AL282. The best results in the table appear in bold.

Models		15-min on Training Set				15-min on Test Set			Variance
		RMSE	MAE	MAPE		RMSE	MAE	MAPE	
k-NNR	uniform	54.544	5.745	5.90		53.361	5.911	6.55	0.65
	distance	33.512	4.308	3.34		53.361	5.911	6.55	3.21
SARIMA		193.554	13.120	30.03		55.820	6.658	8.53	21.5
SVR	linear	54.612	6.040	6.71		49.689	6.046	7.05	0.34
	rbf	57.077	6.441	7.69		51.386	6.310	7.74	0.05
	poly	58.136	6.894	9.29		66.506	7.135	9.47	0.18
LR		54.853	6.195	6.04		52.512	5.786	7.08	1.04
SSNN		52.901	5.706	5.81		47.849	5.794	6.26	0.45
LSTM NN		52.270	5.728	5.89		48.196	5.811	6.30	0.41
AEs		60.041	6.117	6.70		48.326	6.028	6.81	0.11
CNN1		64.040	7.081	9.06		95.915	7.653	9.82	0.76
CNN2		51.772	5.775	6.02		53.829	5.804	6.32	0.30
The Proposed Method		49.794	5.554	5.79		52.644	5.675	**6.20**	0.45

**Table 5:** Experimental results of the proposed method and the baseline methods on link AL281. The best results in the table appear in bold.

Models		15-min on Training Set				15-min on Test Set			Variance
		RMSE	MAE	MAPE		RMSE	MAE	MAPE	
k-NNR	uniform	20.173	3.172	1.88		42.941	5.307	5.45	3.57
	distance	44.663	5.235	5.14		42.941	5.307	5.45	0.31
SARIMA		162.669	12.242	26.63		52.826	6.346	8.02	18.61
SVR	linear	41.994	5.346	5.26		40.253	5.349	5.70	0.44
	rbf	45.094	5.789	6.22		45.056	5.833	6.85	0.63
	poly	48.578	6.071	6.84		49.200	6.128	7.59	0.75
LR		38.789	5.219	5.30		48.141	5.746	6.05	0.75
SSNN		41.290	5.281	4.98		38.737	5.182	5.21	0.23
LSTM NN		41.097	5.196	4.90		37.845	5.112	5.14	0.24
AEs		47.404	5.563	5.58		41.638	5.615	6.13	0.55
CNN1		51.0347	5.90	6.29		45.772	5.968	6.87	0.58
CNN2		40.367	5.166	4.87		42.34	5.154	5.16	0.29
The Proposed Method		39.278	5.081	4.79		41.19	5.024	**5.02**	0.23

**Table 6:** Experimental results of the proposed method and the baseline methods on individual links in terms of MAPE. The best results in the table appear in bold.

LinkID	Kalman Filter	LR	CNN2	LSTM	AES	CNN 1	SSNN	The Proposed Method
AL344	13.89	5.83	6.09	5.68	10.98	18.42	6.03	**5.46**
AL3276	10.87	5.69	5.63	5.37	6.93	13.27	5.53	**5.17**
AL444	8.48	6.02	5.98	6.16	8.38	27.28	6.49	**5.97**
AL2878	16.03	10.34	7.96	8.41	14.1	12.83	8.93	**7.66**
AL2869	13.31	6.85	6.89	7.56	12.7	24.24	7.88	**6.84**
AL2871	13.39	8.58	6.64	6.79	10.64	11.3	7.48	**6.03**
AL2861	10.72	15.83	7.82	7.61	10.66	14.41	7.91	**7.46**
AL2853	8.43	5.08	4.73	5.32	7.3	15.03	5.17	**4.56**
AL2850	8.06	6.12	6.46	6.42	8.83	9.95	6.54	**6.13**
AL2852B	5.31	2.02	1.82	1.8	2.11	4.78	1.94	**1.75**
AL2852A	6.41	5.44	5.32	5.38	5.78	9.28	5.41	**5.27**
AL286	5.17	1.29	1.25	1.26	1.92	5.05	1.52	**1.21**
AL292	4.56	1.42	1.29	2.43	1.93	4.82	2.24	**1.26**
AL283	5.09	2.81	1.94	1.87	2.59	4.78	1.96	**1.9**
AL278	13.34	11.08	10.86	11.82	13.04	15.16	12.12	**10.81**
AL270	5.07	2.75	1.83	1.75	2.4	5.07	2.02	**1.78**
AL265	9.95	6.37	6.11	6.24	8.52	10.17	6.37	**5.9**
AL261A	10.05	6.88	6.77	6.61	8.87	10.86	6.58	**6.54**
AL258A	6.64	5.33	5.72	5.67	6.76	8.63	5.84	**5.46**
AL256	9.85	9.56	7.15	7.0	9.29	9.66	6.92	**6.92**
AL2282	8.05	6.27	6.22	6.1	7.77	9.54	6.37	**6.17**
AL248	8.44	5.39	5.65	5.64	7.11	9.31	5.6	**5.56**
AL241	8.89	6.41	6.19	6.08	6.87	7.84	6.55	**5.96**
AL242	7.59	9.35	6.95	6.84	7.42	10.14	6.95	**6.77**
AL236	8.57	7.66	7.16	7.36	8.1	10.71	7.74	**7.05**
AL2292	9.36	7.98	7.71	7.92	9.35	8.54	7.75	**7.57**
AL2295	7.30	5.46	5.1	5.38	6.37	6.83	5.31	**4.65**
AL237	6.84	6.29	6.32	6.41	7.1	6.84	6.6	**6.08**
AL243	7.94	10.4	5.55	6.26	6.68	12.46	5.77	**5.5**
AL240	7.83	6.74	6.09	6.03	6.86	8.78	6.49	**5.97**
AL254	9.85	8.53	7.72	7.57	9.34	9.93	7.93	**7.48**
AL257A	7.07	6.5	6.2	6.08	7.16	7.63	6.52	**6.08**
AL267	7.50	6.42	5.02	5.36	6.73	7.55	5.15	**4.96**
AL272	3.95	1.95	1.41	1.72	1.72	3.33	1.64	**1.33**
AL279	19.26	16.75	14.89	17.12	19.79	26.38	17.0	**14.04**
AL284	5.88	6.29	6.17	6.1	6.92	8.86	6.54	**6.06**
AL291	4.86	2.	1.26	1.29	2.01	3.9	1.49	**1.20**
AL288	6.24	2.43	1.71	1.74	3.01	5.02	1.83	**1.64**
AL2851A	6.98	5.83	5.8	5.69	6.08	6.59	5.81	**5.76**
AL2851B	9.49	6.11	5.93	6.25	8.41	10.55	6.56	**5.88**
AL2849	8.75	8.6	5.62	6.2	7.78	12.16	5.9	**5.38**
AL298	10.56	6.58	6.07	5.64	8.13	28.13	5.73	**5.39**
AL2862	7.27	6.43	6.19	6.14	7.24	7.75	6.84	**5.7**
AL343	11.26	9.21	5.2	6.44	11.15	9.31	6.11	**5.03**
AL2872	5.42	6.68	4.88	5.02	6.06	9.05	5.66	**4.72**
AL2870	6.58	5.9	5.82	5.95	7.05	9.96	6.17	**5.75**
AL2879	14.13	7.12	6.76	6.62	10.72	26.1	6.87	**6.73**
Mean	**8.73**	**6.61**	**5.70**	**5.88**	**7.59**	**11.03**	**6.038**	**5.50**
